# Construction of a traditional Chinese medicine syndrome-specific outcome measure: the Kidney Deficiency Syndrome questionnaire (KDSQ)

**DOI:** 10.1186/1472-6882-12-73

**Published:** 2012-06-06

**Authors:** Run Qiu Chen, Chit Ming Wong, Tai Hing Lam

**Affiliations:** 1 LKS Faculty of Medicine, The University of Hong Kong, 10 Sassoon Road, Pokfulam, Hong Kong, SAR, China; 2 School of Public Health, LKS Faculty of Medicine, The University of Hong Kong, 21 Sassoon Road, Pokfulam, Hong Kong, SAR, China

## Abstract

**Background:**

Development of Traditional Chinese Medicine (TCM) syndrome-specific outcome measures is needed for the evaluation of TCM syndrome-specific therapies. We constructed a Kidney Deficiency Syndrome Questionnaire (KDSQ) for the evaluation of the common TCM syndromes Kidney-Yin Deficiency Syndrome (KDS-Yin) and Kidney-Yang Deficiency Syndrome (KDS-Yang) in middle-aged women with menopausal symptoms.

**Methods:**

KDS-Yin and KDS-Yang were traditionally defined by expert opinion were validated by exploratory factor analysis (EFA) and structural equation modeling (SEM). Content validity was tested by EFA on a sample of 236 women from a seminar and SEM on another sample of 321 women from a postal survey. Other psychometric properties were tested on 292 women from the seminar at baseline and two systematically selected sub-samples: 54 who reported no changes in discomforts 11–12 days after the baseline and 31 who reported changes in discomforts 67–74 days after the baseline. All participants completed the KDSQ, the Greene Climacteric Scale and the standard 12-item Short Form Health Survey.

**Results:**

The EFA and SEM established the measurement models of KDS-Yin and KDS-Yang supporting content validity of the KDSQ. Internal consistency was good (Cronbach’s Alpha >0.70). Construct validity was supported by theoretically-derived levels of correlation with the established external measures. Test–retest reliability was strong (ICC_agreement_: KDS-Yin, 0.94; KDS-Yang, 0.93). The KDSQ was responsive to changes over time as tested by effect size and longitudinal validity.

**Conclusions:**

The KDSQ was a valid and reliable measure for KDS-Yin and KDS-Yang in Hong Kong Chinese middle-aged women with menopausal symptoms.

## Background

The theory of traditional Chinese medicine (TCM) is established on the basis of ancient Chinese philosophies. TCM considers health is the physiological functions in self-regulation, adaptation to the environment, resistance against pathogens and self-recovery from illness; whereas illness is the dysfunction of these physiological aspects [1]. Guided by TCM theory, practically, an illness is diagnosed and differentiated into the TCM pathogenic patterns or syndromes of yin, yang, excess, deficiency, heat, cold, etc. from patient-reported symptoms together with the signs identified by TCM practitioners’ observation, listening, smelling, and palpation [2]. The syndromes may vary in patients having the same illness because of individual difference in health; and thus are managed by TCM syndrome-specific therapies [1,2].

The efficacy of TCM therapies has been the subject of study for the last few decades. Some authors have attempted to align research design with TCM clinical practice within the constraints of a clinical trial [3,4]; whereas many TCM clinical trials adopt a conventional research design [5]. A study evaluating 19 clinical trials shows the positive effects of ginseng on participants having the conditions of study with the TCM indication of ginseng (Qi deficiency syndrome) but the effects of ginseng were negative on participants having the conditions of study without Qi deficiency syndrome, suggesting TCM clinical trial design aligned with clinical practice is necessary to validate and improve future TCM clinical trials [6].

However, TCM syndromes are defined by expert opinion [2,7,8] and their descriptions are vague and vary in textbooks and references [9]. Also, diagnosis of TCM syndromes is subjective [2] and diagnostic consistency can be as low as about 30% among TCM practitioners [10]. In addition, validated and standardised TCM syndrome-specific instruments have not been developed for outcome assessment in TCM clinical trials; instead, TCM clinical trial outcomes have been assessed by researchers subjectively, or using generic or condition-specific health-related quality of life measures [5]. These practises have impeded interpretation of TCM clinical trial reports. We recently have reported that TCM syndromes can be validated with an evidence-based method [11]; and TCM syndromes may be standardised based on that scientific evidence is a higher form of knowledge than expert opinion and experience [12]. Also, we have incorporated the patient-reported outcome (PRO) approach into designing the evidence-based validation [11] to improve reliability of clinical information [13]. Next, it is interesting to find out whether TCM syndrome-specific PRO instruments can be developed for evaluative use in TCM clinical studies. We have shown that the common TCM syndromes presented in women with menopausal symptoms, Kidney-Yin Deficiency Syndrome (KDS-Yin) and Kidney-Yang Deficiency Syndrome (KDS-Yang) [14], can be validated in Hong Kong Chinese middle-aged women with menopausal symptoms [11]. The present study attempted to construct a Kidney Deficiency Syndrome Questionnaire (KDSQ) and test content validity, internal consistency, construct validity, test-retest reliability and responsiveness of the KDSQ for the evaluation of KDS-Yin and KDS-Yang in Hong Kong middle-aged women with menopausal symptoms.

## Methods

Human research ethics approval was obtained from Institutional Review Board of the University of Hong Kong and Hospital Authority Hong Kong West Cluster and written informed consents were obtained from the study participants.

### The KDSQ

In the validation of KDS-Yin and KDS-Yang study [11], we developed a KDS item reduction list with the symptoms of KDS-Yin and KDS-Yang listed in a TCM diagnosis textbook for Chinese national tertiary education [2], a commonly cited clinical research guideline for diagnosis and assessment of TCM syndromes [7], the World Health Organization (WHO) international standard terminologies on TCM (including TCM syndromes) [8], the Chinese national standard on the diagnosis of TCM syndromes for clinical practice [15], and TCM classic literature [16]. The contemporary textbook and references are written by national or international renowned experts in TCM [2,7,8,15]. The classic literature [16] is recognized to have established the foundation of TCM theory and generally, contemporary TCM literature is written with authors’ interpretation to the classic literature [1].

For content validity, we set inclusion and exclusion criteria to select items into the KDSQ [11]. The items included were those directly related to KDS-Yin and KDS-Yang in middle-aged women. The items excluded were those directly related to syndromes other than KDS-Yin and KDS-Yang; those have weak consensus in the literature (i.e., listed in only one or two of the references); and the signs appeared only in the contemporary literature but not in the classic literature. The items included were given indicative guidelines for severity scores ranging from 0 (absent) to 1 (mild), 2 (moderate) and 3 (severe). Then, we pilot-tested the KDSQ on participants who fulfilled the participant inclusion and exclusion criteria for the present study, and finally three senior TCM experts who had over forty years of experience in TCM teaching, research and practice reviewed the items for appropriateness and completeness.

### Participant recruitment

We recruited Chinese women aged 40–60 years, whose primary residence was in Hong Kong, and who could read and write Chinese for questionnaire evaluation. Women were excluded if they had life-threatening diseases, traumatic injuries or currently taking anti-inflammatory, hormone replacement therapy or Chinese Medicines because inclusion of such women would confound the interpretation of the symptom reporting.

All participants were screened for menopausal symptoms using the 21-item standard Greene Climacteric Scale (GCS) (Hong Kong Chinese) [17] and a 3-item urogenital scale [17,18]. Women who reported either mild, moderate or severe grades of any of the 24 menopausal symptoms were included. All women were also asked to complete the KDSQ and the SF-12 (Chinese [Hong Kong] Standard Version 1.0) [19].

At baseline, we recruited a sample of participants from attendants of a health seminar undertaken for the project in 2005 [11]. These women were recontacted by telephone in the order in which they registered for the seminar to identify [i] a retest subsample of 50 women who reported no changes in their discomforts in the 11^th^ to 12^th^ days after the seminar (a time interval estimated to be long enough for the women to forget how they had previously responded but sufficiently short that their symptoms were unlikely to have changed [20]) and [ii] a follow-up subsample of 25 women who reported changes in their discomforts in the 67^th^ to 74^th^ days after the seminar (a time frame estimated to be long enough for genuine changes to have occurred [20]). These women were invited to attend a clinic consultation where they completed the study instruments, and each woman of the follow-up subsample was asked if she had experienced alleviation or worsening of her discomforts.

We also recruited another sample of participants from a postal survey undertaken for this study in 2007. Women participated in the survey were respondents to a second newspaper article about the research. All the respondents were sent a consent form, the study instruments and a stamped reply envelop.

### Statistical analysis

We tested content validity of the KDSQ in four steps. Of which, the first three steps were also designed for validation of KDS-Yin and KDS-Yang [11] and the final step was intended to test the disagreements of description about the item-domain measurement relationships reviewed in the literature [2,7,8,15,16]. In the first step, exploratory factor analysis (EFA) was used to identify whether the items of KDSQ based on their mutual correlation could be grouped in a pattern which showed the characteristics of KDS-Yin and KDS-Yang as described in the literature [2,7,8,15,16]. The factors were yielded with eigen values exceeding unity and were obtained by Varimax rotation with Kaiser Normalization [21]. We also tested whether a control item selected from those listed in the literature [2,7,8,15,16] but were not included in the KDSQ could be grouped with the factor(s) of KDS-Yin or KDS-Yang.

In the second step, based on the factors yielded we constructed latent tree models [22] to show the symptoms (the observed variables) that measure the domains (the latent variables) of KDS-Yin and KDS-Yang. Then, we added additional measurement relationships to the models to reveal those disagreements of description were reviewed in the literature [2,7,8,15,16] which were also supported by the EFA findings: some symptoms were identified by the EFA to measure one domain and also had a relatively large factor loading from other domain(s) indicating these symptoms to a lesser extent might also measure these other domain(s).

In the third step, we attempted to validate these measurement models by structural equation modelling (SEM) on the survey sample. These models would be validated if the comparative fit index (CFI) and the incremental fit index (IFI) were equal to or greater than .90, the root mean square error of approximation (RMSEA) was less than or equal to .05, and 90% confidence interval (CI) of RMSEA ranged from 0 to 0.08 [22].

Finally, we attempted to strengthen these latent measurement models by trimming symptom-domain measurement relationships fulfilling the following criteria: those measurement relationships were justifiable by some but disagreed by other of the literature [2,7,8,15,16]; as tested by SEM the factor loadings (regression weights) of those measurement relationships were identified to be small and not significantly different from zero at the 0.05 level [20]; and after trimming those measurement relationships the models could still be validated by SEM based on indices listed in the third step and were justifiable by the literature [2,7,8,15,16]. The scaling structure of KDS-Yin and KDS-Yang was defined based on these measurement models, and the scoring method for the domains of KDS-Yin and KDS-Yang was the sum of scores reported by women to the symptoms within the domains.

We further evaluated the KDSQ as follows. Internal consistency was tested by Cronbach α coefficient on the seminar sample [23]. An intraclass correlation coefficient in absolute agreement (two-way random effects model) was computed on these interval-like Likert scale (normal, mild, moderate, and severe) data for test-retest reliability on the retest subsample [24]. A coefficient value of 0.70 was used as a standard for group-level analysis for these tests [25]. A hypothesis that the scores of KDS-Yin and the scores of KDS-Yang were strongly correlated with the scores of the GCS, but negatively and moderately correlated with the scores of SF-12 was tested for construct validity using Spearman’s correlation on the seminar sample. The negative correlation was assumed since higher symptom scores would indicate poorer generic health. A moderate or weaker correlation was expected since the SF-12 is a generic health survey whereas the GCS is a menopause-specific scale. Finally, whether the KDSQ was responsive to change over time was determined by the effect size [(baseline mean - follow-up mean)/SD at baseline] and the longitudinal validity, which was tested by Spearman correlation coefficients on the follow-up subsample.

Data were processed by SPSS for Windows 16.0 and AMOS 16.0. Missing data were handled by AMOS. AMOS uses maximum likelihood imputation to estimate means and intercepts for missing data, which has been shown to have the least bias [26].

## Results

Of the 311 women who attended the seminar, 294 were invited to complete the KDSQ, the GCS and the urogenital scale, and the SF-12 after excluding 17 women who were on medications (n = 16) or younger than 40 (n = 1). We excluded two more women who missed most data in the KDSQ (n = 2). The seminar sample included 292 women. From them, 54 women were identified for evaluating test-retest reliability and another 31 women were identified for testing responsiveness. Of the 31 women, 24 responded to all items including low libido and vaginal dryness of the KDSQ. Of these 24 women, 19 reported alleviation of discomforts and 5 reported worsening of discomforts. From the 292 women, 236 were included for the EFA after excluding women who did not respond to the item on libido due to absence of sex partners (n = 54) and women who missed some data in their KDSQ (n = 2). Except marital status and the small percentages of women who had hysterectomy and bilateral ovariectomy, demographic characteristics were similar between the 236 women and those excluded from the EFA (n = 54) (Table 1), indicating the non-response bias would not have significant impact on the EFA. A total of 435 women who responded to the postal survey were sent the survey documents. Eight surveys were not delivered due to incorrect mailing addresses and 335 women returned their questionnaires, giving a response rate of 78.5%. Fourteen participants were excluded from the analysis because their questionnaires had missing data (n = 5), blank or marked with “not suitable” (n = 4), or completed by women younger than 40 years (n = 2) or older than 60 years (n = 3). The postal survey sample included 321 women. These women were slightly younger, more were in paid employment, less were classified as postmenopausal and more were not married than were the women in the seminar sample who were included to the EFA (p < 0.05), whereas BMI and education were similar (P > 0.05) (Table 1). All these women were identified to have suffered from menopausal symptoms as measured by the 21-item GCS and the 3-item urogenital scale.

**Table 1:** Sociodemographic characteristics of the samples

	**Seminar (EFA) (n = 236)**	**Seminar (Excl.) (n = 54)**	**Survey (n = 321)**
Mean age (years)	49.7 ± 4.8	48.9 ± 4.9	48.2 ± 5.6
BMI (kg/m^2^)	22.2 ± 2.8	21.9 ± 2.9	22 ± 2.8
Menopausal status			
Premenopausal	25.8%	25.9%	47%
Perimenopausal	33.5%	29.6%	20.9%
Postmenopausal	33.1%	29.6%	24.6%
Hysterectomy*	4.7%	13%	2.8%
Ovariectomy-bilateral*	2.9%	1.9%	4.7%
Marital status*			
Now married	90.3%	16.7%	70.7%
Not married	9.7%	83.3%	29.3%
Educational level			
Primary	14.0%	13.7%	11.2%
Secondary	69.5%	68.8%	63.2%
Tertiary	16.5%	17.5%	25.6%
Employment			
Employed	39.8%	38.8%	74.1%
Retired/house wife/unemployed	60.2%	61.2%	25.9%

Of the seminar sample, participants included to the EFA and those excluded were similar (p > 0.05) except where marked by * (p < 0.05). Between the seminar sample (EFA) and the survey sample, BMI and education were similar (p > 0.05) but age, menopausal status, marital status and employment were different (p < 0.05).

### The KDSQ

**Figure 1:**
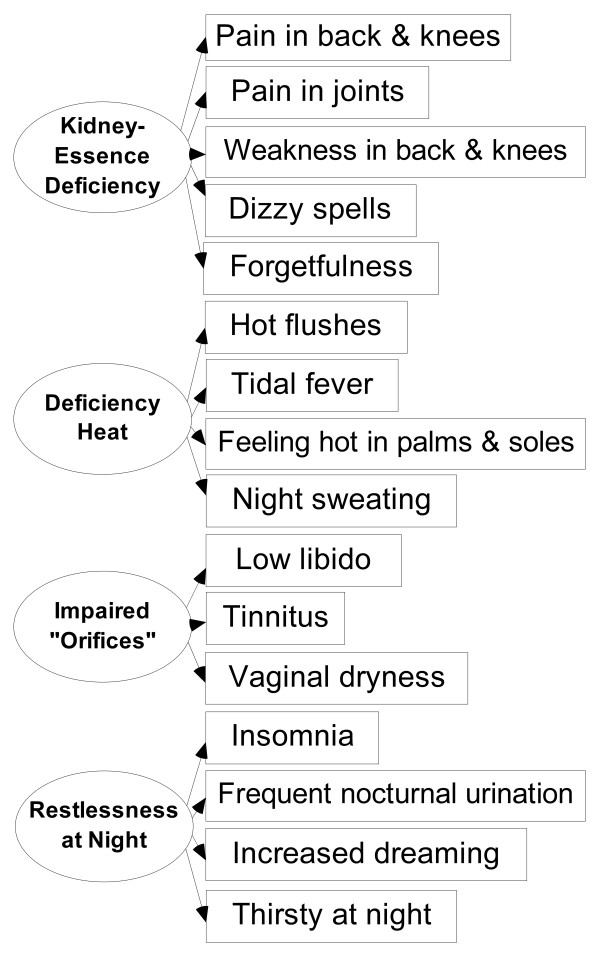
**The measurement model of KDS-Yin.** “Thirsty at night” was identified to measure “Kidney-Essence deficiency” by the EFA was trimmed off by the SEM; whereas this symptom that added to measure “restlessness at night” was validated by the SEM. Of the KDSQ 24 items, 16 measure KDS-Yin, 9 are common items to KDS-Yin and KDS-Yang.

**Figure 2:**
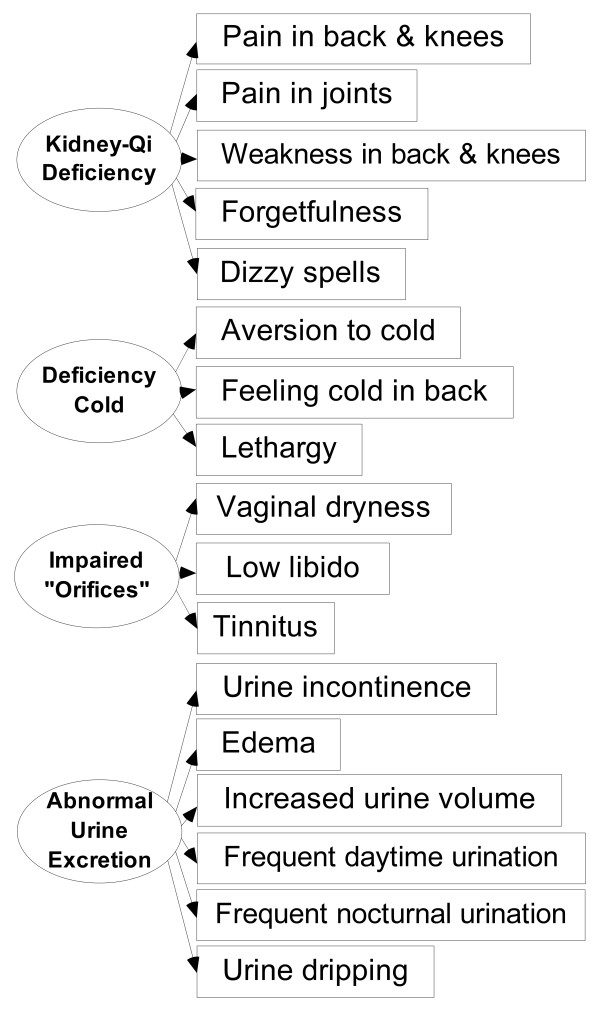
**The measurement model of KDS-Yang.** The EFA defined five domains but the SEM merged the fifth domain “abnormal water metabolism” to “abnormal urine excretion” for they were found to be highly correlated (>0.90), and this merge was justifiable by the theory. “Urine dripping” and “dizzy spells” were identified by the EFA to measure “Kidney-Qi deficiency” and “deficiency cold”, respectively, were trimmed off by the SEM; whereas these symptoms added to measure “abnormal urine excretion” and “Kidney-Qi deficiency”, respectively, were validated by the SEM. Of the KDSQ 24 items, 17 measure KDS-Yang, 9 are common items to KDS-Yin and KDS-Yang.

From all the symptoms and signs described in the literature [2,7,8,15,16], we included 39 symptoms to the KDS item reduction list by excluding the items appear only in children (retarded growth and development), men (impotence, nocturnal emission, premature ejaculation, infertility) and women of reproductive stage (infertility, oligomenorrhea), and the signs of pulse, tongue, and facial complexion that do not appear in the classic literature [16] (KDS-Yin: reddened tongue with scanty coating, rapid and fine pulse; KDS-Yang: pale facial complexion, pale tongue, deep and weak pulse). Of the 39 items, we excluded another nine items that are rather directly related to the lung (cough, asthmatic breathing); the heart (fearfulness); the spleen (loose stools, bowel incontinence); cold invasion (stiffness in the low back or knees); excess heat (constipation, high libido); excessive water intake (clear urine). The 30-item KDS item reduction list was pilot tested on ten women who were recruited by convenient sampling. Based on the pilot test, we excluded six items usually appear in old aged women and men with KDS (hearing impairment or loss, hair turning grey and wither, hair loss, dried teeth, loose teeth, teeth fall). Finally, we included 24 items in the KDSQ for the measurement of KDS-Yin (Figure 1) and KDS-Yang (Figure 2) in middle-aged women. Three senior TCM experts approved the KDSQ (Additional file 1).

### Psychometric properties

In the first step, EFA on the seminar sample (n = 236) was shown with Kaiser-Meyer-Olkin Measure of Sampling Adequacy greater than 0.6 [27]: KDS-Yin, 0.84; KDS-Yang 0.83; and Bartlett’s test of Sphericity: KDS-Yin, p < 0.0001; KDS-Yang, p < 0.0001. The EFA identified four symptom factors for KDS-Yin (Figure 1) and five symptom factors for KDS-Yang (Figure 2). With reference to the theory [2,7,8,15,16], the four factors of KDS-Yin were found to correspond to Kidney-Essence deficiency, deficiency heat, impaired Kidney orifices and restlessness at night; whereas the five factors of KDS-Yang correspond to Kidney-Qi deficiency, deficiency cold, impaired Kidney orifices, abnormal water metabolism, and abnormal urine excretion. The item “loose stools” listed only in the textbook [2] for the diagnosis of KDS-Yang was added as a control to the KDSQ, but was not found to be grouped to the domains of KDS-Yang by the EFA indicating this control item was independent from the domains of KDS-Yang. More details on the EFA had been reported as part of a study on the symptom characteristics of KDS-Yin and KDS-Yang elsewhere [28].

In the second step, we constructed the initial measurement models of KDS-Yin and KDS-Yang with the factors yielded by the EFA. Then, we added 13 and 14 additional symptom-domain measurement relationships within these models of KDS-Yin and KDS-Yang, respectively, based on the disagreements in the literature [2,7,8,15,16] and the factor loadings yielded by the EFA, producing two multi-collinear measurement models in which some symptoms measured more than one domains of KDS-Yin or KDS-Yang. In the third step, these multi-collinear models could be validated by SEM on the postal survey sample as reported elsewhere [11].

In the final step, we found that the factor loadings of most of the added additional measurement relationships (KDS-Yin, 12; KDS-Yang, 12) and few measurement relationships as defined by the EFA (KDS-Yin, 1; KDS-Yang, 2), as indicated in the figure legends, were small (<0.2) and not significant at the 0.05 level. These measurement relationships were weak and trimmed off according to the exclusion criteria set at the Methods section. The measurement relationships retained in Figures 1 and 2 their factor loadings ranged from 0.23 to 0.71 for KDS-Yin and from 0.24 to 0.74 for KDS-Yang, and all were significantly different from zero at the 0.05 level. The measurement model of KDS-Yin (Figure 1) tested by the SEM was found to be fit to the data (CFI, 0.90; IFI, 0.90; RMSEA, 0.04; and a 90% CI of RMSEA, 0.02–0.06), whereas the measurement model of KDS-Yang (Figure 2) was marginally fit to the data (CFI, 0.83; IFI, 0.84; RMSEA, 0.06; and a 90% CI of RMSEA, 0.04 - 0.07). Content validity of the KDSQ was determined by these exploratory, confirmatory and model strengthening tests.

The KDSQ items were found to have good internal consistency in measuring the important domain concepts: Kidney-Essence deficiency and deficiency heat of KDS-Yin, and Kidney-Qi deficiency and deficiency cold of KDS-Yang; and have weaker homogeneity in measuring the other less important domain concepts (Table 2).

**Table 2:** Internal consistency

	**Cronbach’s Alpha**	
	**Baseline**	**(Sample A)**
KDS-Yin	0.81	(n = 235)
Kidney-Essence deficiency*	0.76	(n = 292)
Deficiency heat*	0.72	(n = 292)
Impaired Kidney orifices	0.61	(n = 235)
Restlessness at night	0.48	(n = 292)
KDS-Yang	0.81	(n = 235)
Kidney-Qi deficiency*	0.76	(n = 292)
Deficiency Cold*	0.70	(n = 292)
Impaired Kidney orifices	0.61	(n = 235)
Abnormal urine excretion	0.56	(n = 292)

The symptoms had good internal consistency in taping the concepts of KDS-Yin and KDS-Yang and their important domains * (>.70).

Test-retest reliability was good for the domains of KDS-Yin and KDS-Yang. ICC _agreement_ was 0.94 for KDS-Yin and was 0.89 for KDS-Yang, and the range was 0.87–0.93 for the domains of KDS-Yin and KDS-Yang.

Construct validity was supported by that the items of KDS-Yin and the items of KDS-Yang measured what they were supposed to measure. Spearman’s correlation coefficients (*r*_s_) of the GCS were 0.79 with KDS-Yin and 0.81 with KDS-Yang indicating strong correlations (p < 0.01), whereas the coefficients of KDS-Yin were -0.52 with the physical component score (PCS) and −0.43 with the mental component score (MCS) of SF-12, and KDS-Yang were -0.60 with CS and -0.51 with MCS, indicating expectedly weaker and negative correlations (p < 0.01). Responsiveness of the domains of KDS-Yin and KDS-Yang to change was supported by the effect sizes calculated on 19 of the 24 women who reported alleviation of discomforts during the period of 67–74 days (Table 3). Also, the changes in discomforts in this follow-up sample (n = 24) measured by the GCS were strongly correlated with those measured by KDS-Yin (*r*_s_ =0.71, p < 0.001) and moderately correlated with those measured by KDS-Yang (*r*_s_ =0.40, p = 0.051), supporting the longitudinal validity of the KDSQ.

**Table 3:** The effect size of KDS-Yin and KDS-Yang and their domains

	**ES**		**ES**
KDS-Yin	0.81	KDS-Yang	0.69
Kidney-Essence deficiency	0.84	Kidney-Qi deficiency	0.84
Deficiency heat	0.14	Deficiency cold	0.24
Impaired Kidney orifices	0.46	Impaired Kidney orifices	0.46
Restlessness at night	0.76	Abnormal urine excretion	0.49

ES, effect size: 0.2, small; 0.5, medium; 0.8 large.

## Discussion

We have constructed a TCM syndrome-specific measure the KDSQ and found the KDSQ is a valid and reliable tool for the measurement of KDS-Yin and KDS-Yang in Hong Kong Chinese middle-aged women with menopausal symptoms.

Scientific evidence is recognised to be a higher form of knowledge than expert opinion [12]. We tested expert opinions in our construction of the KDSQ for expert opinion that is subjective nature varies among experts. TCM syndromes are defined by expert opinion in plain language with domain changes and symptoms. Similar to what is reported by Birch et al. [9], we found that the description of KDS-Yin and KDS-Yang was vague and varied in the literature despite experts had attempted to standardize TCM syndromes for decades [2,7,8,15]. Content validity of the KDSQ could not be established by expert opinion. Therefore, after excluding those KDS items which were not related to KDS in middle-aged women (e.g., retarded growth and development, impotence), we tested the KDSQ for content validity by the pilot-test, the EFA exploratory test, and the SEM confirmatory and model-strengthening tests on different samples of participants. The EFA exploratory and the SEM confirmatory tests also constitute a novel approach for evidence-based validation of TCM syndromes, and for which we have reported that TCM syndromes may be validated and standardized based on scientific evidence for education, research and practice [11].

How to properly measure TCM clinical trial outcomes has been explored in recent years. Some authors developed an outcome measure for TCM syndromes in people with hepatitis C virus [29]. In this study, 38 TCM syndromes for hepatitis were identified from seven sources of published literature including one clinical trial, one case study and five textbooks. The authors reduced the 38TCM syndromes into 17 for outcome measure after revealing some similarity in both name and symptom cluster with reference to a TCM practical dictionary, suggesting the descriptions of TCM syndromes in hepatitis were vague and varied in the literature. However, whether the symptom cluster could be validated as well as validity and reliability of the 17 syndromes for outcome measure in the patients with hepatitis were not reported.

Other authors developed a generic Cold-Heat Pattern Questionnaire as an adjunct diagnostic tool for Cold and Heat TCM syndromes [30], and also a generic quantitative measure of Yin scores and Yang scores for diagnostic evaluation of Yin and Yang TCM syndromes [31]. In TCM, Yin and Yang, Cold and Heat are rather qualitative concepts [1,2,16]. Patients with a medical condition their symptoms can be assessed against the qualities of Yin (i.e., cool, dark, quietness, etc.) and Yang (i.e., warmth, bright, motion, etc.), cold (i.e., cold feeling, preference to warmth, etc.) and heat (i.e., hot sensation, preference to cool, etc.) for a gross diagnosis of Yin or Yang, and Cold or Heat syndromes, respectively [2]. Conceptually, content validity can only be established for condition-specific tools for diagnostic measurement of Yin or Yang, and Cold or Heat syndromes because different medical conditions manifest with different symptoms. Also, some authors have attempted to establish a Yang deficiency constitution questionnaire [32] or develop a Chinese quality of life instrument [33] using the Delphi method. The Delphi method was not used in the construction of the KDSQ because it is an iterative process for consensus-building of expert opinions only among a panel of experts and also, the Delphi researchers may subjectively synthesize questionnaire responses from experts that could induce researcher bias [34].

There are limitations of the present study. We reviewed the classic literature that was recognized to have built the foundation of TCM theory [16] and the contemporary literature that had been most frequently cited in TCM publications on education, research and practice [2,7,8,15]. We considered these references were representative of the TCM theory for the study of KDS-Yin and KDS-Yang. In future studies, we need to develop guidelines for the selection of references from TCM literature contributed by scholars of the last two thousand years and the present time. Also, the sample size of the pilot-test was small (n = 10) and that could be increased to reduce bias. The participants of the seminar and the survey were women who were bothered by menopausal symptoms and were interested in having a free TCM consultation, and that we may test the KDSQ more rigorously on community samples of women with menopausal symptoms. Cultural variation of menopausal symptoms is well reported; but there is little literature on that of KDS symptoms. Cautiousness should be taken in generalizing the validity and reliability of the KDSQ to middle-aged women of other ethnic backgrounds without prior testing.

We constructed the KDSQ with a PRO design by excluding the signs on facial complexion, tongue and pulse that require TCM practitioners’ diagnosis. The exclusion was made because the signs were not described in the classic literature [16], the symptoms were found to measure the domains of KDS-Yin and KDS-Yang [2,7,8,15], and diagnosis of the signs is an important source of low diagnostic consistency [10]. The signs may be included to the construction of TCM instruments when reliable methods for objective diagnosis of the signs are available in the future. Also, the KDSQ is designed to be an outcome measurement tool, but for use as a diagnostic devise more rigorous tests are warranted.

Internal consistency of the KDSQ items was relatively weak in measuring “impaired Kidney orifices”, “restlessness at night” and “abnormal urine excretion”; which may be subject to further study. In contrast, internal consistency was good for Kidney-Essence deficiency and Kidney-Qi deficiency which are important domains of KDS-Yin and KDS-Yang, respectively [1,2,16]; and their importance was supported by that these two domains individually accounted for about 25% of the variance in the EFA data set and that of other domains was less than 10%. Internal consistency was also good for deficiency heat and deficiency cold which are most important for differential diagnosis of KDS-Yin and KDS-Yang [1,2,16]; and their importance was supported by a discriminant analysis studying the symptom characteristics of KDS-Yin and KDS-Yang [28]. The deficiency heat symptoms and deficiency cold symptoms of the KDSQ are mainly those of menopausal vasomotor symptoms [17]. A community survey shows that intensity of the vasomotor symptoms is lower than that of menopausal psychological and somatic symptoms in Hong Kong Chinese middle-aged women, and that of the vasomotor symptoms is significantly lower in Hong Kong Chinese middle-aged women than in Dutch middle-aged women [18].

Consistently, our participants reported relatively mild deficiency heat symptoms or deficiency cold symptoms at baseline test and the magnitudes of change over the period of 66–74 days were relatively small. These may have contributed to the small effect sizes of the deficiency heat domain of KDS-Yin and the deficiency cold domain of KDS-Yang. On the other hand, the rest of the KDSQ domains were found to have about medium to large effect sizes (Table 3), suggesting that the KDSQ could detect changes.

Kidney deficiency causes permanent cessation of menstrual periods in middle-aged women is documented in TCM classic and contemporary literature [14,16]. We have shown evidence supporting the TCM theory on KDS and the menopause [11]. In contrast, some authors have challenged that Kidney deficiency causes menopause is a product of modernization of TCM by copying estrogen deficiency of menopause in biomedicine during the China Cultural Revolution [35,36]. The authors surveyed a sample of London middle-aged women using a general symptom check list consisting of menopausal symptoms and other symptoms (about one third of the total items) not related to menopause or Kidney deficiency in middle-aged women, and the survey did not exclude participants who might have concurrent medical conditions. The authors performed an EFA on the survey data and interpreted factors from the EFA into TCM patterns that could not reflect the treatments of Kidney deficiency [36]. However, we do not find a subjective expert opinion-based interpretation of EFA on the symptoms that may have been confounded by concurrent illnesses can dispute the TCM theory on Kidney deficiency and the menopause. We believe the present study has important implications on TCM research and development. As reported in medical literature, psychometrically sound measures are commonly used in clinical research. Condition-specific measures have been shown to have better responsiveness to change and discriminative ability than generic health measures and hence can provide more accurate outcome measurement of trial intervention [37,38]. Similarly, TCM syndrome-specific measures are expected to improve responsiveness and discriminative ability of TCM outcome measurement.

## Conclusions

The KDSQ is a valid and reliable measure for KDS-Yin and KDS-Yang in Hong Kong Chinese middle-aged women with menopausal symptoms. TCM syndrome-specific measurement tools may be developed for outcome measurement in TCM clinical research.

## Competing interests

The authors declare that they have no competing interests.

## Authors’ contributions

RQC contributed to the design, implementation, and analysis of the study. THL and CMW contributed to the design and analysis of the study. RQC drafted the manuscript, THL and CMW revised the manuscript, and all approved the final version. All authors read and approved the final manuscript.

## Pre-publication history

The pre-publication history for this paper can be accessed here:

http://www.biomedcentral.com/1472-6882/12/73/prepub

## Supplementary Material

Additional file 1 The Kidney Deficiency Syndromes Questionnaire. Click here for file

## References

[B1] Wu DX Textbooks for general tertiary education of chinese medicine: principles of chinese medicine [In Chinese] 2002 Shanghai: Shanghai Scientific and Technical Publishers

[B2] Zhu WF Fei SF Yang MQ Textbooks for general tertiary education of chinese medicine: diagnosis of chinese medicine [In Chinese] 1994 Shanghai: Shanghai Scientific and Technical Publishers

[B3] Bensoussan A Tally N Hing M Treatment of irritable bowel syndrome with Chinese herbal medicine JAMA 1998 280 1585 1589 10.1001/jama.280.18.1585 9820260

[B4] Sung JJY Leung WK Ching JYL Agreements among traditional Chinese medicine practitioners in the diagnosis and treatment of irritable bowel syndrome Aliment Pharmacol Ther 2004 20 1205 1210 10.1111/j.1365-2036.2004.02242.x 15569124

[B5] Davis SR Briganti EM Chen RQ The effects of Chinese medicinal herbs on postmenopausal vasomotor symptoms of Australian women: a randomised controlled trial Med J Aust 2001 174 68 71 11245505 10.5694/j.1326-5377.2001.tb143156.x

[B6] Yan J Engle VF He YX Study designs of randomized controlled trials not based on Chinese medicine theory are improper Chin Med 2009 4 3 10.1186/1749-8546-4-3 19243625 PMC2663767

[B7] Zheng SY, Ren DQ Guidelines for clinical research on new preparation of Chinese herbal medicines [In Chinese] 2002 Beijing: Chinese Medicine Science and Technology Publishing House 385 389

[B8] WHO WHO International Standard Terminologies on Traditional Medicine. WHO Library Cataloguing in Publication Data 2007

[B9] Birch S Sherman K Zhong Y Acupuncture and low-back pain: traditional Chinese medical acupuncture differential diagnoses and treatments for chronic lumbar pain J Altern Compl Med 1999 5 415 425 10.1089/acm.1999.5.415 10537241

[B10] Zhang GG Singh B Lee WL Improvement of agreement in TCM diagnosis among TCM practitioners for persons with the conventional diagnosis of rheumatoid arthritis: effect of training J Altern Compl Med 2008 14 4 381 386 10.1089/acm.2007.0712 18576921

[B11] Chen RQ Wong CM Cao KJ Lam TH An evidence-based validation of traditional Chinese medicine syndromes Compl Ther Med 2010 18 199 205 10.1016/j.ctim.2010.05.036 21056843

[B12] Portney LG Watkins MP Norwalk CT 1. Introduction: A Concept of Research Foundations of Clinical Research: Applications to Practice 1993 1 Appleton & Lange 3 16

[B13] Doward LC McKenna SP Defining patient-reported outcomes Value Health 2004 7 S1 S4 S8 15367236 10.1111/j.1524-4733.2004.7s102.x

[B14] Yuankai L Chinese medicine gynecology 1964 Shanghai: Shanghai Sciences Publishing House [in Chinese]

[B15] The National Technology Bureau, The National Standard of The People’s Republic of China. The Diagnostic Terminologies and Pathogenic Patterns for The Clinical Practice of Chinese Medicine The parameters in the diagnosis of kidney-deficiency syndrome and sub-syndromes [in Chinese] 1997 Beijing: Standard Press of China

[B16] Chen ZX, Song GM Compilation of ten classics of Chinese medicine: Huangdi’s internal classic 1995 Beijing: Xueyuan Publishing [In Chinese]

[B17] Chen RQ Davis SR Wong CW Lam TH Validity and cultural equivalence of the standard Greene Climacteric Scale in Hong Kong Menopause 2010 17 3 630 635 20130493 10.1097/gme.0b013e3181ca0adb

[B18] Lam PM Leung TN Haines C Chung TKH Climacteric symptoms and knowledge about hormone replacement therapy among Hong Kong Chinese women aged 40-60 years Maturitas 2003 45 99 107 10.1016/S0378-5122(03)00090-2 12787968

[B19] Lam CLK Tse EYY Gandek B Is the standard SF-12 Health Survey valid and equivalent for a Chinese population? Qual Life Res 2005 14 2 539 547 10.1007/s11136-004-0704-3 15892443

[B20] Streiner DL Norman GR Health measurement scales: a practical guide to their development and use 2003 3 New York: Oxford University Press

[B21] Factor analysis: Factor Analysis Rotation 2003 SPSS 12.0 for Windows

[B22] Garson GD Structural Equation Modelling. Statnotes: Topics in Multivariate Analysis 2008 http://www2.chass.ncsu.edu/garson/pa765/statnote.htm. Assessed in June

[B23] Aday LA Designing and conducting health surveys 1989 San Francisco: Jossey-Bass

[B24] McGraw KO Wong SP Forming inferences about some intraclass correlation coefficients Psychol Meth 1996 1 30 46

[B25] Nunally JC Bernstein IR Psychometric theory 1994 3 New York: McGraw-Hill

[B26] Byrne BN Structural equation modeling with AMOS 2001 Rahwah: Lawrence Erlbaum Associates 296 297

[B27] MacCallum RC Widaman KF Zhang SB Hong SH Sample size in factor analysis Psychol Meth 1999 4 1 84 99

[B28] Chen RQ Wong CM Cao KJ Lam TH Symptoms characteristics of Kidney-Yin deficiency and Kidney-Yang deficiency in Hong Kong Chinese midlife women J Altern Compl Med 2008 14 5 457 460 10.1089/acm.2007.7202 18532896

[B29] Berle CA Cobbin D Smith N Zaslawski C A novel approach to evaluate traditional Chinese medicine treatment outcomes using pattern identification J Altern Compl Med 2010 16 4 357 367 10.1089/acm.2009.0367 20374102

[B30] Ryu H Lee HJ Kim HG Kim JY Reliability and validity of a cold-heat pattern questionnaire for traditional Chinese medicine J Altern Compl Med 2010 16 6 663 667 10.1089/acm.2009.0331 20569034

[B31] Langevin HM Badger GJ Povolny BK Davis RT Johnston AC Sherman KH Kahn JR Kaptchuk TJ Yin scores and Yang scores: a new method for quantitative diagnostic evaluation in Traditional Chinese Medicine research J Altern Compl Med 2004 11 2 389 395 10.1089/107555304323062392 15165421

[B32] Su YC Chen LL Lin JD Lin JS Huang YC Lai JS BCQ+: a body constitution questionnaire to assess Yang-Xu part I: establishment of a first final version through a Delphi process Forsch Komplementmed 2008 15 327 334 19142042 10.1159/000175938

[B33] Leung KF Liu FB Zhao L Fang JQ Chan K Lin LZ Development and validation of the Chinese quality of life instrument Health Qual Life Outcome 2005 3 26 10.1186/1477-7525-3-26 PMC1090607 15833138

[B34] Garson GD Delphi Method 2008 http://faculty.chass.ncsu.edu/garson/PA765/delphi.htm. Assessed in June

[B35] Scheid V Traditional Chinese medicine—what are we investigating? The case of menopause Compl Ther Med 2007 15 54 68 10.1016/j.ctim.2005.12.002 PMC2233879 17352972

[B36] Scheid V Ward T Tuffrey V Comparing TCM textbook descriptions of menopausal syndrome with the lived experience of London women at midlife and the implications for Chinese medicine research Maturitas 2010 66 4 408 416 10.1016/j.maturitas.2010.03.021 20444560

[B37] Brazier JE Harper R Munro J Walters SJ Snaith ML Generic and condition-specific outcome measures for people with osteoarthritis of the knee Rheumatology 1999 38 870 877 10.1093/rheumatology/38.9.870 10515649

[B38] McTaggart-Cowan HM Marra CA Yang Y Brazier JE Kopec JA FitzGerald JM The validity of generic and condition-specific preference-based instruments: the ability to discriminate asthma control status Qual Life Res 2008 17 3 453 462 10.1007/s11136-008-9309-6 18274882

