# Supplementary material for: Construction of a traditional Chinese medicine syndrome-specific outcome measure: the Kidney Deficiency Syndrome questionnaire (KDSQ)
Source: BMC Complement Altern Med. 2012 Jun 6;12:73. doi: 10.1186/1472-6882-12-73 (PMC3479037; doi:10.1186/1472-6882-12-73)
Supplement: Additional file 1 — The Kidney Deficiency Syndromes Questionnaire. [file 1472-6882-12-73-S1.pdf]

The Kidney Deficiency Syndromes Questionnaire (KDSQ)

（Please √ the ONE that suits you the most ）

Part I	Questions	√	About your urine in the past four weeks	
1.1	How many times do you wake up to urinate during the night?	0	0～1 time	
		1	2～3 times	
		2	4～5 times	
		3	6 times or above	
1.2	How frequent do you urinate during the day?	0	Once every 2 hours or longer intervals	
		1	Once every 1-2 hour intervals	
		2	Once every 30 minute to 1 hour intervals	
		3	Once in less than 30 minute intervals.	
1.3	Is there any dripping after urination?	0	No, urine is clear with no dripping.	
		1	Yes, there are a few urine drips before clearing.	
		2	Yes, urine drips for a while before clearing.	
		3	Yes, urine drips and hardly clear.	
1.4	Do you experience urine incontinence during the day?	0	No, not at all.	
		1	Sometimes, when I am tired.	
		2	Yes, when I have the urge to urinate.	
		3	Yes, anytime. It seems that I do not have any control.	
1.5	What do you think of your urine volume?	0	It is as usual.	
		1	It is slightly more than usual.	
		2	It is moderately more than usual.	
		3	It is a lot more than usual.	
**Part II**	**Questions**	√	**About your sexual activity in the past four weeks**	
2.1	Do you experience reduced libido?	0	No, I do not experience any change in libido.	
		1	Yes, I have desire for sex but sometimes difficult to become aroused and enjoy.	
		2	Yes, I have little desire and enjoyment from sex.	
		3	Yes, I have no desire for sex and I do not enjoy having sex.	
		NA	I do not know. I don't have a sex partner in the past four weeks.	
2.2	Do you experience vaginal dryness?	0	No, it feels about the same as it does usually.	
		1	Yes, it feels a little more dry than usual.	
		2	Yes, it feels quite dry sometimes.	

		3	Yes, it feels very dry.	
Part III	**Questions**		**About your general health in the past four weeks**	
3.1	Do you feel energetic?	0	Yes, I feel energetic.	
		1	No, I feel tired after work, but I recover soon after resting.	
		2	No, I often feel tired, and need a long rest to recover.	
		3	No, I feel very tired and can not recover after resting.	
3.2	Do you feel swelling in the face, hands and feet?	0	No, not at all.	
		1	Yes, sometimes I feel swelling in the face, hands or feet.	
		2	Yes, I often feel swelling in the face, hands or feet.	
		3	Yes, I feel swelling and distending in the face, hands or feet.	
3.3	Are you averse to wind and cold?	0	No, not at all.	
		1	Yes, but I do not need to wear more clothing.	
		2	Yes, but I am fine after wearing more clothing.	
		3	Yes, I wear more clothing but still feel averse to cold.	
3.4	Do you feel cold or chills in the back?	0	No, not at all.	
		1	Yes, sometimes but I do not need to wear more clothing.	
		2	Yes, but I feel fine after wearing more clothing.	
		3	Yes, I feel cold in the back after wearing more clothing.	
3.5	Do you experience hot flushes in the cheeks?	0	No, not at all.	
		1	Yes, I experience occasional mild flushes in a week.	
		2	Yes, I have several moderate hot flushes in a week.	
		3	Yes, I have frequent severe hot flushes in a week.	
3.6	Do you experience dizzy spells?	0	No, not at all.	
		1	Yes, occasionally for a short period of time.	
		2	Yes, often with fluctuating severity, and I feel better with my eyes shut.	
		3	Yes, often severe as if in a boat the sky is moving, and I do not feel any better with my eyes shut.	
3.7	Do you experience feverish feeling that comes and goes like tidal waves?	0	No, not at all.	
		1	Yes, sometimes and mild, comes and lasts for a short time.	
		2	Yes, frequent and moderate, lasting for 30 minutes to an hour.	
		3	Yes, every day and severe, lasting for an hour or longer.	

3.8	Do you have hot sensations in the chest, palms and the soles of your feet?	0	No, not at all.	
		1	Yes, occasionally and mild.	
		2	Yes, often with fluctuating severity, or occurs intermittently.	
		3	Yes, often severe and I feel more comfortable in the cold.	
3.9	Do you sweat during sleep?	0	No, not at all.	
		1	Yes, sometimes, very little and my clothing is not wet.	
		2	Yes, there are wet patches in my clothing when I wake up.	
		3	Yes, my clothing is wet all over when I wake up.	
3.10	Is the sleep quality and quantity good?	0	Yes, I sleep well.	
		1	No, I sleep 5-6 hours and sometimes difficult to fall asleep.	
		2	No, I may sleep 3-4 hours and often difficult to fall asleep.	
		3	No, I may sleep 3 hours or less and difficult to fall asleep.	
3.11	Do you tend to forget things that happened recently?	0	No, not at all.	
		1	Yes, I am not remembering things as well as used to be.	
		2	Yes, I tend to forget things happened recently.	
		3	Yes, I forget things happened recently and in the past.	
3.12	Do you experience high pitch ringing in the ears?	0	No, not at all.	
		1	Yes, relatively mild and some days I don't hear it.	
		2	Yes, some days severe and some days mild.	
		3	Yes, I constantly hear the sound.	
3.13	Do you experience weakness in the back and knees?	0	No, not at all.	
		1	Yes, I feel the weakness when I am standing up from the squatting position or tired, but I feel better after a rest.	
		2	Yes, I am difficult to stand up from the squatting position or walking the stairs.	
		3	Yes, I am difficult to stand up from the squatting position, sitting, standing, or walking.	
3.14	Do you experience pain in the joints other than the back and knees?	0	No, not at all.	
		1	Yes, mild.	
		2	Yes, moderate.	
		3	Yes, severe.	
3.15	Do you experience pain in the back and knees?	0	No, not at all.	
		1	Yes, mild and I may not feel the pain sometimes.	
		2	Yes, moderate and when I am tired, or recover after a rest.	

		3	Yes, frequent and severe, and restrict the joint movement.	
3.16	Do you feel thirsty when wake up at night or in the morning?	0	No, not at all.	
		1	Yes, mild and I don't need to drink water.	
		2	Yes, moderate and thirsty reduces with drinking water.	
		3	Yes, severe and thirsty does not reduce with drinking water.	
3.17	Do you dream at night sleep?	0	No, not at all.	
		1	Yes, occasional dreaming but doesn't affect my sleep quality.	
		2	Yes, almost every night but doesn't affect my sleep quality.	
		3	Yes, every night and affects my sleep quality.	

(The questions correspondingly ask for the symptoms in Figures 1 & 2:
1.1, Frequent nocturnal urination; 1.2, Frequent daytime urination; 1.3, Urine dripping; 1.4, Urine incontinence; 1.5, Increased urine volume; 2.1, Low libido; 2.2, Vaginal dryness; 3.1, Lethargy; 3.2, Edema; 3.3, Aversion to cold; 3.4, Feeling cold in back; 3.5, Hot flushes; 3.6, Dizzy spells; 3.7, Tidal fever; 3.8, Feeling hot in palms and soles; 3.9, Night sweating; 3.10, Insomnia; 3.11, Forgetfulness; 3.12, Tinnitus; 3.13, Weakness in back & knees; 3.14, Pain in joints; 3.15, Pain in back & knees; 3.16, Thirsty at night; 3.17, Increased dreaming.)
